# Characteristics of *PoVIN3*, a Key Gene of Vernalization Pathway, Affects Flowering Time

**DOI:** 10.3390/ijms232214003

**Published:** 2022-11-13

**Authors:** Yuying Li, Can Wang, Qi Guo, Chengwei Song, Xiaohui Wang, Lili Guo, Xiaogai Hou

**Affiliations:** 1 College of Agronomy/Tree Peony, Henan University of Science and Technology, Luoyang 471023, China; 2 Luoyang Academy of Agriculture and Forestry Sciences, Luoyang 471023, China

**Keywords:** tree peony, *PoVIN3*, flowering regulation, expression analysis, functional analysis

## Abstract

The tree peony (*Paeonia* section *Moutan* DC.) is the candidate flower in China, with abundant germplasm resources and high ornamental value. However, the short and concentrated flowering period severely restricted the improvement of the economic value of tree peonies. Based on the full-length transcriptome database of tree peonies, the *PoVIN3* (GenBank ID: OP341879), involved in the flowering regulation of tree peonies were identified and cloned for the first time. The *PoVIN3* was also characterized by bioinformatics methods, quantitative real-time PCR (qRT-PCR), and the establishment of a transgenic system. The expression levels of *PoVIN3* in seven different petals developmental stages were the highest at the initial flowering stage of the variant cultivar of *Paeonia ostii* ‘Fengdan,’ the initial decay stage of the normal flowering *Paeonia ostii* ‘Fengdan,’ and the half opening stage of the late flowering *Paeonia suffruticosa* ‘Lianhe.’ Tissue-specific expression analysis showed that the relative expression levels of *PoVIN3* were the highest in sepals of both normal flowering *Paeonia ostii* ‘Fengdan’ and the late flowering *Paeonia suffruticosa* ‘Lianhe,’ and the highest expression was in stamens of early flowering mutant *Paeonia ostii* ‘Fengdan.’ In addition, the flowering time of pCAMBIA2300-*PoVIN3* transgenic plants was significantly earlier than that of the wild-type, indicating that PoVIN3 could promote plant flowering. The results provide a theoretical basis for exploring the role of *PoVIN3* in the regulation of flowering in tree peonies.

## 1. Introduction

The tree peony (*Paeonia* section *Moutan* DC.) is a famous traditional flower in China. It has a high ornamental, cultural, and economic value with big flowers, bright colors, and elegant appearance, and is renowned as a symbol of Chinese civilization [1]. The research on ornamental characteristics of tree peony has made continuous progress from breeding varieties to cultivation technology, which has been widely cultivated worldwide, and now contains as many as 2200 ornamental varieties [2]. However, the flowering time of tree peonies is short and concentrated, and a widespread social consensus and regret with the short period of flowering and viewing. Under natural conditions, it takes 50–60 days from flower bud initiation to petal decay, and the flowering time is 3–5 days for a single flower (a single flower from the full blooming stage to the decay stage) and 10–15 days for the various group (the flowering period from the first to the last flower of the same variety). Most tree peonies are middle-flowering varieties, with smaller numbers of early− and late−flowering varieties [3,4]. The flowering period determines the commercial value of tree peonies [5,6]. Consequently, a thorough understanding of the genetic basis and molecular regulatory mechanisms related to the regulation of tree peony flowering will help to meet the demand of people for prolonged ornamental traits in tree peonies.

Flowering is an important process for higher plants to achieve the transition from vegetative to reproductive growth. The MADS−box transcription factor named the *FLOWERING LOCUS C* (*FLC*) is a key gene in the *Arabidopsis thaliana* flowering regulatory network and plays a pivotal role in flowering regulation [7]. There are many genes controlling flowering in the autonomous pathway and vernalization pathway, and their functions were realized by regulating the expression of *FLC*. *FLC* inhibited flower formation mainly by acting on the flowering integration genes *FLOWERING LOCUS T* (*FT*) and *SUPPRESSOR of OVEREXPRESSION of CO 1* (*SOC1*). *FT* and *SOC1* integrated signals from flowering pathway signals and are called floral pathway integrators. *FLC* inhibited their expression by binding to the CArG box of the *SOC1* promoter and part of the *FT* first intron (containing the CArG box), respectively [8,9], thereby inhibiting floral meristem determining genes and thus delaying flower formation. Vernalization mainly causes changes in the chromatin structure of *FLC* so as to relieve its inhibitory effect on flowering [10].

*VERNALIZATION INSENSITIVE 3* (*VIN3*) has been identified as an essential gene for triggering vernalization and inhibiting *FLC*, and the induction of *VIN3* expression was the earliest event of vernalization. Together with *VERNALIZATION 1* (*VRN1*) and *VERNALIZATION 2* (*VRN2*), *VIN3* forms a complex that acts on histones on *FLC* chromatin to deacetylate and methylate them, thereby inhibiting *FLC* expression, and the plant exhibits early flowering [11,12,13]. At low temperatures, *VIN3* inhibited *FLC* expression by recognizing the time of low−temperature treatment during vernalization and acting together with *VRN1*, *VRN2,* and *VRN5* [14]. *VIN3* had the characteristic of sensing the time course of low temperature. The expression of *VIN3* could only be induced under long-term low−temperature treatment, and *VIN3* could not be detected after the plant returned to normal temperature, but *FLC* was still inhibited. Therefore, the role of *VIN3* is to identify the time of low−temperature treatment in the vernalization process and establish the suppression of the expression of *FLC* [13]. *VIN3* encodes a PHD−finger protein, which can be involved in the methylation and deacetylation of nucleosome histones, causing the remodeling of chromatin structure [15]. PHD domain is a class of conserved C4CH3 (Cys4−His−Cy3) structure composed of about 60 amino acid residues, belonging to the “cross-brace” zinc finger protein family, which can change the spatial structure of chromatin to cause gene silencing [16,17]. Upon induction of *VIN3*, which is essential for PHD−PRC2 nucleation, H3K9 and H3K27 on the histones of *FLC* chromatin were dimethylated and trimethylated, thus showing genetic silencing and plants showing early flowering [18,19].

According to previous reports on the VIN gene family in *Arabidopsis thaliana* and *Poaceae*, this study focused on the key gene *VIN3* in upstream of the vernalization pathway in the flowering network. The *PoVIN3* related to flowering regulation was cloned from a tree peony for the first time. The sequence of *PoVIN3* was analyzed by biological software, and the expression of *PoVIN3* in tree peonies was detected by qRT−PCR, which provided a theoretical basis for studying the molecular mechanism of tree peony flowering regulation.

## 2. Results

### 2.1. Cloning and Sequence Analysis of PoVIN3

**Figure 1:**
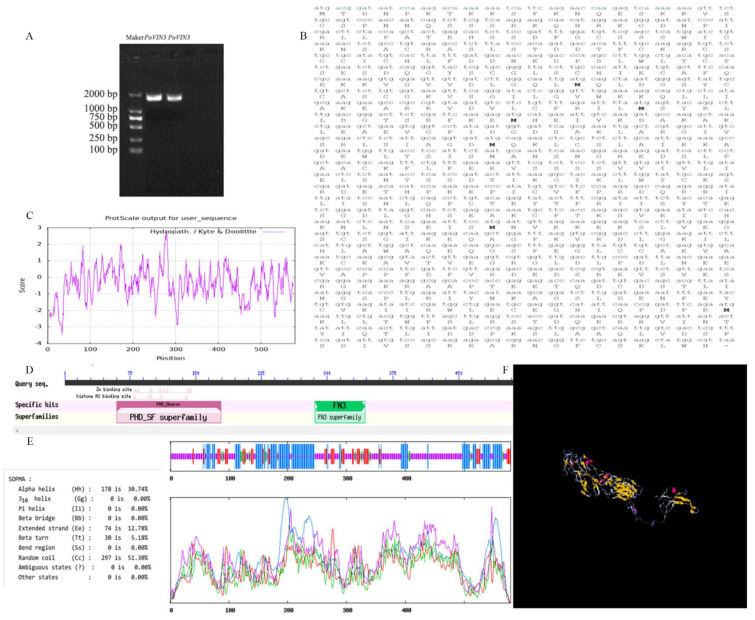
Cloning and bioinformatics analysis of *PoVIN3*. (**A**) PCR amplified product of *PoVIN3*, and M means DNA maker DL2000. (**B**) Nucleotide sequence and its encoded amino acid sequence. (**C**) Hydrophilic analysis of *PoVIN3*−encoded protein. (**D**) Analysis of conserved domain of *PoVIN3*. (**E**) Secondary structure prediction of *PoVIN3*−encoded protein. (**F**). Tertiary structure prediction of *PoVIN3−*encoded protein.

The target fragment was obtained by using the petal cDNA of FD as a template and named *PoVIN3*. The gene sequence was submitted to GenBank (OP341879) in NCBI. The complete open reading frame (ORF) of *PoVIN3* is 1740 bp in total (Figure 1A,B) and encodes a polypeptide with 579 amino acids. The predicted molecular mass of *PoVIN3−*encoded protein was found to be 64.76 kD, and the isoelectric point (pI) was 7.62. The instability coefficient was 49.66 and was classified as unstable. In addition, the total average hydrophilicity was −0.400, indicating that the protein was hydrophilic (Figure 1C). The analysis of conserved domains showed that the protein contained two domains, including the PHD-SF (Accession: PFAM07227) superfamily region present at amino acids 60–179 and the 286–343 amino acids containing the FN3 (Accession: Smart100060) superfamily conserved domain (Figure 1D). The predicted protein secondary domain shows that the protein amino acids mainly exist as alpha helices (178 amino acids), extended strands (74 amino acids), random coils (297 amino acids), and beta helices (30 amino acids) (Figure 1E). Furthermore, according to the template 1zlgA model, the confidence score (C−score) value is −2.03 (Figure 1F).

### 2.2. Phylogenetic Tree Analysis

**Figure 2:**
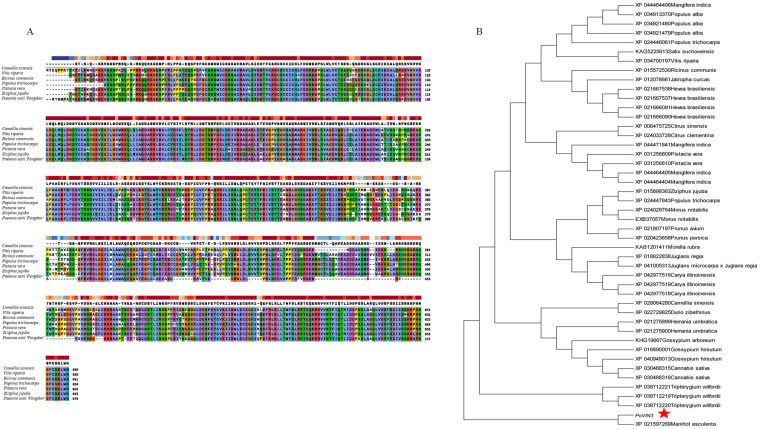
Multiple sequence alignment (**A**) and phylogenetic relationship (**B**) of *PoVIN3*−encoded protein and *VIN3*−encoded protein from other plant species. The red asterisk represented the *PoVIN3*−encoded protein.

In order to study the evolutionary relationship between *PoVIN3* of tree peony and *VIN3* in other plants, *VIN3* coding amino acid sequences of different plants were selected from NCBI for comparative analysis. The *PoVIN3*−encoded protein was obtained from *Camellia sinensis* (accession number: XP_028064280), *Ricinus communis* (accession number: XP_015572530), *Ziziphus jujuba* var. *spinosa* (accession number: XP_048320875), *Vitis Riparia* (accession number: XP_034700197), *Pistacia Vera* (accession number: XP_031256809), and *Populus Trichocarpa* (accession number: XP_024446061) had high homology of 100%, 98%, 98%, 98%, and 96%, respectively (Figure 2A).

The phylogenetic tree of 28 different plants was constructed, including *Populus_trichocarpa* (XP_024446061, XP_024447843), *Ziziphus jujube* (XP_015898363), *Pistacia vera* (XP_031256809, XP_031256810), *Populus alba* (XP_034921479, XP_034921480, XP_034913370), *Actinidia chinensis* var. *chinensis* (PSS01860), *Gossypium hirsutum* (XP_016690001, XP_040949013), *Camellia sinensis* (XP_028064280), *Salix suchowensis* (KAG5225613), *Ricinus communis* (XP_015572530), *Morella rubra* (KAB1201411), *Prunus avium* (XP_021807197), *Gossypium arboretum* (KHG19667), *Prunus persica* (XP_020423658), *Mangifera indica* (XP_044471841, XP_044464405, XP_044464404, XP_044464406), *Tripterygium wilfordii* (XP_038712221, XP_038712219, XP_038712220), *Carya illinoinensis* (XP_042977516, XP_042977519, XP_042977518), *Hevea brasiliensis* (XP_021667538, XP_021667537, XP_021666091, XP_021666090), *Morus notabilis* (XP_024029764, EXB37057), *Juglans regia* (XP_018822838), *Citrus sinensis* (XP_006475725), *Citrus clementine* (XP_024033728), *Juglans microcarpa × Juglans regia* (XP_041005313), *Jatropha curcas* (XP_012078661), *Herrania umbratica* (XP_021275899, XP_021275900), *Manihot esculenta* (XP_021597269), *Cannabis sativa* (XP_030488315, XP_0304883160), *Durio zibethinus* (XP_022729825), and *Vitis riparia* (XP_034700197). Compared with the *PoVIN3*−encoded protein, the relationship between the *VIN3*−encoded protein of *Vitis riparia* and that of *Salix suchowensis* was close. VIN3−like protein (XP_021597269) from *Manihot esculenta* was phylogenetically closest to *PoVIN3*-encoded protein (Figure 2B).

### 2.3. Expression Pattern of PoVIN3

**Figure 3:**
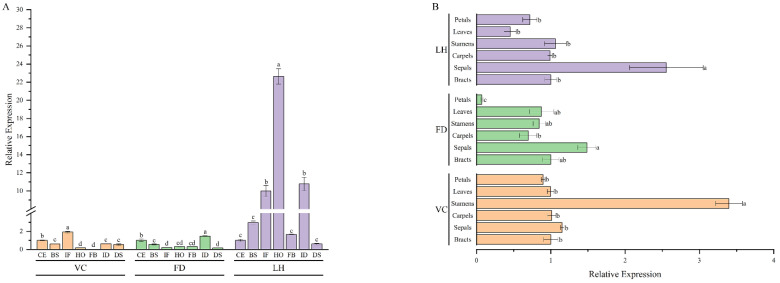
Expression patterns of *PoVIN3* in tree peony. (**A**) The relative expression levels of *PoVIN3* in different developmental stages. (**B**) The relative expression levels of *PoVIN3* in different tissues. Error bars represent standard error (SE). Different lowercase letters indicate significant differences at *p* < 0.05.

In order to determine the spatiotemporal expression patterns of *PoVIN3*, the expression levels were investigated by qRT−PCR within samples taken from various tissues and petals at different developmental stages of VC, FD, and LH. The qRT−PCR results demonstrated that the expression levels of *PoVIN3* in LH were significantly higher than that in VC and FD at different developmental stages (CE, BS, IF, HO FB, ID, and DS) (Figure 3A). In VC, *PoVIN3* had a high expression level in the early stage of flower development (CE, BS, and IF), and the expression level was significantly higher in the IF stage (*p* < 0.05). The expression levels were significantly lower in the HO and FB stages and increased again in the decline process (ID and DS) but still lower than that in the early stage (Figure 3A). During FD flower development to flower blooming, *PoVIN3* expression levels decreased first and then increased, reached a significantly lower level at IF, and then increased sharply at ID, reaching a significantly higher level (*p* < 0.05) (Figure 3A). The *PoVIN3* expression levels of LH were different from that of the former two. With the progress of flower development, *PoVIN3* expression levels gradually increased and reached a significantly higher level at HO (*p* < 0.05). The expression level of *PoVIN3* decreased abruptly in the FB stage and increased abruptly in the ID stage. When the petals decayed, the *PoVIN3* expression level decreased to a significantly lower level (*p* < 0.05) (Figure 3A).

*PoVIN3* was expressed in all tissues collected from the three tree peony cultivars, including bracts, sepals, carpels, stamens, leaves, and petals. However, the expression level in the leaves of FD was at the lower limits of detection (Figure 3B). Moreover, the expression levels of *PoVIN3* were significantly higher in the sepals of FD and LH across all tissue samples (*p* < 0.05) (Figure 3B). It is worth noting that the expression of *PoVIN3* in VC of early flowering tree peonies was mainly concentrated in stamens, while the lowest expression level was found in petals (Figure 3B).

### 2.4. Subcellular Localization

**Figure 4:**
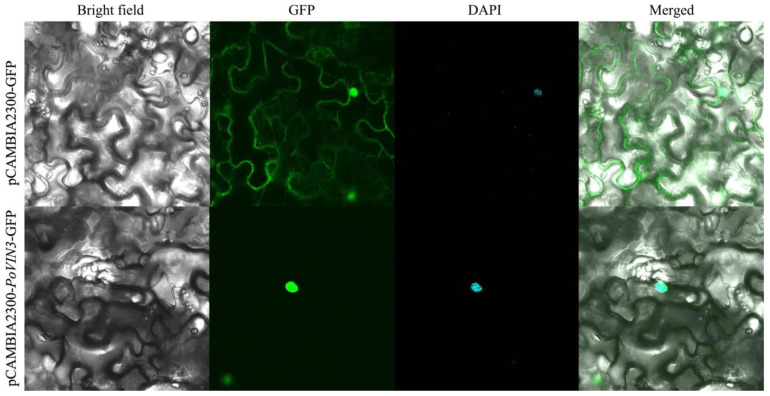
Subcellular localization of GFP fusions of *PoVIN3*.

Prediction of subcellular localization suggested that *PoVIN3*−encoded protein was localized in the nucleus. A fusion expression vector pCAMBIA2300−*PoVIN3*−GFP was transiently transformed in *N. benthamiana* to determine the subcellular localization of *PoVIN3*−encoded protein, while the pCAMBIA2300−GFP was a control. Subsequently, confocal scanning microscopy was used to observe the *Agrobacterium tumefaciens*−infected leaves of *N. benthamiana* containing the gene of interest. This study found that the pCAMBIA2300−GFP emitted green fluorescence throughout the cells under the excitation of 488 nm wavelength, while the strong fluorescence signals were distributed in the nucleus for the pCAMBIA2300−*PoVIN3*−GFP, consistent with the prediction. Thus, indicating that *PoVIN3* was indeed nuclearly localized (Figure 4).

### 2.5. Ectopic Expression of PoVIN3 Accelerate Flowering in Arabidopsis Thaliana

**Figure 5:**
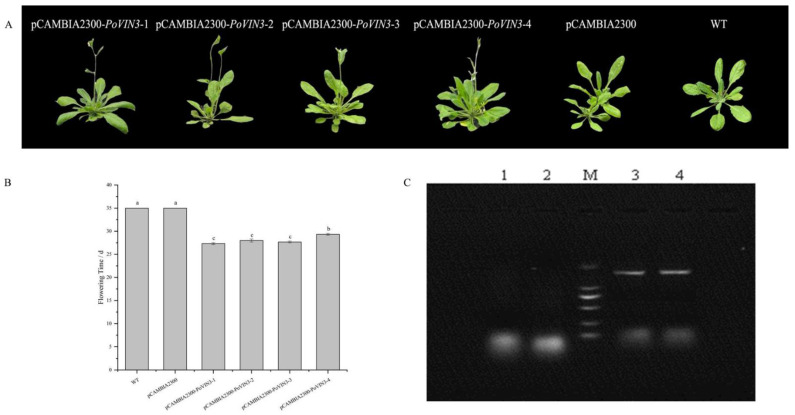
The phenotype and PCR amplification of *Arabidopsis thaliana* transformed with *PoVIN3*. (**A**) The phenotypes of the 4−week−old transgenic *Arabidopsis thaliana* and WT plants. (**B**) The flowering time of transgenic *Arabidopsis thaliana* and WT plants. The standard error (SE) is indicated by error bars. Different lowercase letters (a–c) indicate significant differences at *p* < 0.05. (**C**) PCR assay of *PoVIN3* in WT (1), pCAMBIA2300 plants (2), overexpression *Arabidopsis thaliana* plants (3–4), and M means DNA maker DL2000.

The spatiotemporal expression pattern of *PoVIN3* suggested that it might play a key role in the regulation of flowering time and flower development in plants. To prove that *PoVIN3* can promote the early flowering of *Arabidopsis thaliana*, four groups of transgenic *Arabidopsis thaliana* plants over-expressing pCAMBIA2300−*PoVIN3*−1, pCAMBIA2300−*PoVIN3*−2, pCAMBIA2300−*PoVIN3*−3, and pCAMBIA2300−*PoVIN3*−4, respectively, were generated (Figure 5A). All four groups of transgenic plants showed an early flowering phenotype compared with the control plants transformed with an empty vector and WT plants, and the flowering time was significantly advanced by 5.67–7.67 days (*p* < 0.05) (Figure 5B). At the same time, 40−day−old leaves of T_2_ plants were harvested, and the results of RT−PCR analysis of *PoVIN3* also proved that the transgenic lines obtained specific amplified bands of the expected size; by contrast, no specific bands were detected in either WT or pCAMBIA2300 plants (Figure 5C). These results suggest that *PoVIN3* plays an important role in the flowering process of plants under long-day conditions.

### 2.6. pCAMBIA2300-PoVIN3 Overexpressed Tree Peony Petals

**Figure 6:**
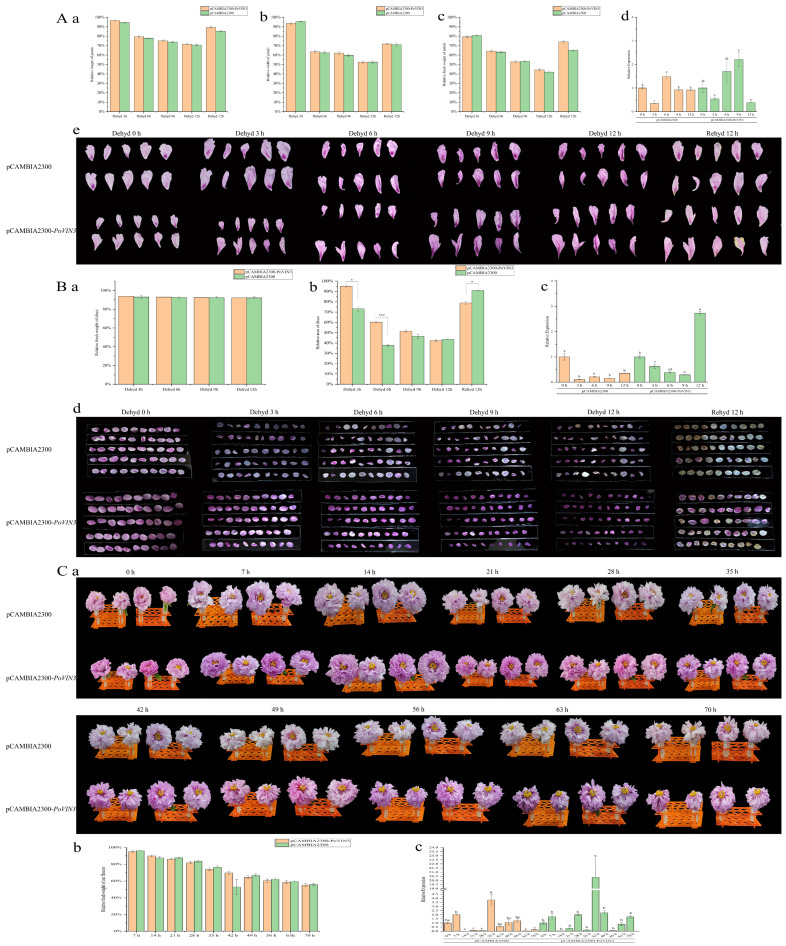
Transient expression of *PoVIN3* was overexpressed in tree peony petals, discs, or fresh-cut flowers. (**A**) Tree peony petals were infiltrated with *Agrobacterium tumefaciens* containing pCAMBIA2300 control and pCAMBIA2300-*PoVIN3* for dehydration (0, 3, 6, 9, 12 h) and rehydration for 12h. The relative length (**a**), relative width (**b**), relative fresh weight (**c**), relative expression (**d**), and phenotype (**e**) of petals were analyzed. (**B**) Phenotype and recovery of *PoVIN3*-overexpressed petal discs. The relative fresh weight (**a**), relative area (**b**), relative expression (**c**), and phenotype (**d**) of petal discs were analyzed. (**C**) Overexpression infection in fresh cut flowers of tree peony. The phenotype (**a**), relative fresh weight (**b**), and relative expression (**c**) of fresh cut flowers were analyzed. Error bars represent standard error (SE). Different lowercase letters (a–d) indicate significant differences at *p* < 0.05, * means *p* < 0.05, and *** means *p* < 0.001.

To test the possible role of *PoVIN3* in tree peony petals, we overexpressed PoVIN3 in tree peony petals by transient expression technique. During dehydration, *Paeonia suffruticosa* ‘Luoyanghong’ petals infected with the pCAMBIA2300−*PoVIN3* overexpression vector generally showed a slightly longer length than petals infected with pCAMBIA2300 empty vector, but there was no significant difference (*p* > 0.05) (Figure 6A(a,e)). After 12 h of dehydration, the relative width of petals infected with pCAMBIA2300−*PoVIN3* was about 52.42% ± 1.12% of its initial value (Figure 6A(b,e)), and the relative fresh weight of all petals was about 44.24% ± 1.02% of its initial value, 5.18% as compared with the empty vector control (41.95% ± 1.06%) (*p* > 0.05) (Figure 6A(c,e)). During 12 h of rehydration, petals infected with pCAMBIA2300−*PoVIN3* returned to 89.51% ± 7.28% (the relative length of petals), 71.73% ± 8.25% (the relative width of petals), and 73.93% ± 1.16% (the relative fresh weight of petals) of their initial value. However, only 85.26% ± 0.89% (the relative length of petals), 71.01% ± 1.54% (the relative width of petals), and 65.14% ± 1.19% (the relative fresh weight of petals) of their initial value were infected with pCAMBIA2300 empty vector. Similarly, qRT−PCR showed that there was an extremely high expression in the petals of *Paeonia suffruticosa* ‘Luoyanghong’ after 9 h dehydration (Figure 6A(d)).

The effects of gene overexpression and dehydration were also tested on petal discs. In the process of dehydration, the relative fresh weight of petal discs infected with the pCAMBIA2300−*PoVIN3* overexpression vector was slightly higher than that infected with pCAMBIA2300 empty vector, but there was no significant difference (*p* > 0.05) (Figure 6B(a,d)). Moreover, this phenomenon was also confirmed by the relative area (Figure 6B(b,d)). However, after 12h of rehydration, petal discs infected with pCAMBIA2300 empty vector could recover to 91.02% ± 0.73% of their initial value, which was significantly higher than that infected with overexpressed bacteria (*p* < 0.05) (Figure 6B(b)). The relative expression of *PoVIN3* in the overexpressed infected petal discs during dehydration was higher than that in the empty vector control and reached the peak at 12 h after dehydration (Figure 6B(c)).

Notably, in the infection process of fresh cut flowers of *Paeonia suffruticosa* ‘Luoyanghong,’ it was found that the fresh cut flowers infected with pCAMBIA2300−*PoVIN3* reached FB 7 h earlier than that of empty vector infection, which was consistent with the results obtained from the stable expression of *Arabidopsis thaliana*, indicating that *PoVIN3* may advance the flowering time of tree peony (Figure 6C(a)), but there was no significant difference in the relative fresh weight between them (*p* > 0.05) (Figure 6C(b)). The relative expression level of *PoVIN3* in the fresh-cut flowers of *Paeonia suffruticosa* ‘Luoyanghong’ was detected. It was found that the relative expression level of *PoVIN3* in the petals infected with overexpression reached the highest at 42 h after transient expression, and a certain amount of *PoVIN3* was still expressed at 70 h (Figure 6C(c)).

## 3. Discussion

Vernalization of plants is of great significance to the development of economic benefits, and many plants need to be exposed to the cold for a period of time, that is, undergo the vernalization process, to induce flowers, as this indicates the end of winter and avoids mortality from premature flowering in the fall [20]. Based on the full−length transcriptome database of tree peonies previously constructed in our laboratory, genes related to vernalization were screened. Primers were designed to clone the target gene. *PoVIN3* (GenBank ID: OP341879) was cloned from a tree peony for the first time by RT−PCR, which was also the first time to obtain key genes in the vernalization pathway from ornamental plants were, and its expression characteristics and functions were preliminarily studied.

Sequence analysis showed that the vernalization−related gene *PoVIN3* had high homology with the corresponding gene in *Camellia sinensis*. *PoVIN3* contains two domains, namely, the PHD−SF superfamily and the FN3 superfamily. This was consistent with the structure of *VIN3* in *Arabidopsis thaliana* [13], *Brassica oleracea* L. var. *capitata* L. [21], and *Brassica compestris* ssp. *chinensis* var. *pupurea Hort.* [22]. PHD−finger protein is one of 14 known zinc−binding motifs, which are found in more than 400 eukaryotic proteins and are highly conserved during evolution [23]. Three homologous genes of *VIN3* were cloned from *Triticum monococcum* L., namely *TmVIL1(VIN3*−*like1)*, *TmVIL2*, and *TmVIL3*, and also four *VEL* family genes (*VIN3* homologous gene family) were obtained from *Oryza Sativa* (*OsVIL1*, *OsVIL2*, *OsVIL3*, and *OsVIL4*). They all encode proteins with the zinc−binding motif of C4HC3 (CYS4−His−Cys3), which is conserved in the PHD domain [15]. Studies have shown that the PHD domain is involved in many functions, including protein methylation, acetylation, phosphorylation, ubiquitination, and so on [15,24]. In this study, the results of subcellular localization showed that *PoVIN3*−encoded protein was localized in the nucleus, indicating that *PoVIN3* had the function of encoding protein. Therefore, we speculated that *PoVIN3* might act on *FLC* through the PHD domain, thereby shutting down *FLC* expression and inhibiting *FLC* function.

In this study, it was found that the expression trend of *PoVIN3* was not the same among the three tree peony varieties, and the overall expression trend was that the late−flowering *Paeonia suffruticosa* ‘Lianhe’ was higher than the early−flowering and normal flowering *Paeonia ostii* ‘Fengdan’ at different stages of flower development, indicating that *PoVIN3* may have variety specificity. The results were similar to the expression pattern of *BcVIL1* (*VIN3*−*LIKE1* belongs to the *VIN3* family gene) in non−heading Chinese cabbage [25]. *VIN3* is a protein produced only in cold winters, and its cold−inducing ability is related to the degree of vernalization [26]. Numerous experiments have shown that *VIN3* plays a role in mediating proper vernalization in *Arabidopsis* [27,28]. In the process of mango flowering induction, it was also confirmed that *VIN3* could be expressed or up−regulated only after prolonged low−temperature induction [29]. Although the changes of *PoVIN3* expression patterns during cold vernalization were not involved in this study, the expression of *PoVIN3* in tree peonies from flower formation to decay was demonstrated, which provided a new idea for the study of this gene. In addition, we demonstrated that there were significant differences in the expression of *PoVIN3* in various organs and tissues of tree peonies. As expected, *PoVIN3* expression was abundant in various floral organs and tissues. These results suggested that P*oVIN3* remains active during floral organ differentiation and growth. In contrast to our results, the expression of rice *VIN3* was high in leaves, moderate in seedlings, roots, and presbyteria, and low in stems and young flowers [30]. The lower expression of *VIN3* in floral tissues may be due to the fact that rice plants do not require vernalization, unlike plants such as *Arabidopsis*, cereal, and tomato, which show a strong flowering response to vernalization [31,32].

Previous studies have confirmed that the function of *VIN3* was to inhibit the expression of *FLC*, a key gene for vernalization, so as to promote flowering [33,34]. The tree peony is a traditional Chinese flower, and the research on the mechanism of regulating tree peony flowering has always been the focus of researchers [35,36]. In this study, *PoVIN3* was cloned from the petals of *Paeonia ostii* ‘Fengdan’ at the full blooming stage. In order to investigate the function of *PoVIN3*, an important gene in the vernalization pathway that regulates flowering, we constructed a pCAMBIA2300−*PoVIN3* overexpression vector and transformed it into *Arabidopsis Thaliana*. According to the statistics of the flowering time of wild−type and transgenic *Arabidopsis thaliana* under long−day conditions, the flowering time of the transgenic *Arabidopsis thaliana* was significantly advanced by 5.67–7.67 days (*p* < 0.05) (Figure 5B). It was speculated that *PoVIN3* might promote flowering. Based on the current experimental studies, further studies on the relationship between *PoVIN3* and other transcription factors and its role in the vernalization process of peony should be the focus of future work. Due to the large reference genome and many repetitive sequences in tree peony, the tree peony ontology cannot be used for functional verification of genes at present. Therefore, the model plant *Arabidopsis Thaliana* was selected to verify the possible function of *PoVIN3* in this experiment. This method is often used for functional gene verification in tree peonies [37]. In addition, the methods for verifying the gene function of *Arabidopsis thaliana* transformation are also common in plants with large genomes and imperfect genetic transformation systems, such as herbaceous peonies [38] and cotton [39]. Therefore, it is reliable to select *Arabidopsis Thaliana* as the receptor for verifying *PoVIN3* function in this study.

The progress of genetic transformation in tree peonies has been hampered by long-term regeneration schemes, which are required for successful ontological genetic transformation in tree peonies, and the low efficiency of regeneration systems further aggravates the establishment of genetic transformation systems in tree peonies. At present, the identification of functional genes in tree peonies rely on model plants such as *Arabidopsis thaliana,* but it takes a long time to analyze the function of introduced transgenes [40]. Therefore, using transient expression of the gene to determine gene function was an effective alternative method while avoiding the problems of low transformation efficiency and long generation time of transgenic plants. The establishment of efficient and rapid gene function analysis methods in plants has become a research hotspot in recent years [41]. A transient expression system could overexpress or silence target genes in plant cells in a short period of time, which greatly accelerates the process of gene function research. In rose [42], kiwifruit [43], lily [44], grape [45], strawberry [46], and petunia [47]. The transient expression system was established in plants by the *Agrobacterium tumefaciens*−*mediated* method. In this study, the transient transformation system of tree peony overexpression was also established by *Agrobacterium tumefaciens*. The results laid a foundation for the verification of the function of *PoVIN3* in tree peony and also provided research ideas and a technical basis for the verification of other important functional genes in tree peonies.

Based on the above results, *PoVIN3* may play a role in regulating flowering time. This is the first time to clone and obtain the cDNA ORF coding region of vernalization−related gene *PoVIN3* in tree peony, which has great reference significance for further study of vernalization-mediated flowering pathway in tree peony and can lay a foundation for improving the ornamental and economic value of tree peony.

## 4. Materials and Methods

### 4.1. Plant Materials and Growth Conditions

For the analysis of gene expression patterns, plant materials were obtained from the tissues (including bracts, sepals, carpels, stamens, leaves, and petals) of a variant cultivar of *Paeonia ostii* ‘Fengdan’ that exhibits earlier flowering (VC), a normal flowering *Paeonia ostii* ‘Fengdan’ (FD), and a late flowering *Paeonia suffruticosa* ‘Lianhe’ (LH). All the species used as test material was planted in the experimental field of Henan University of Science and Technology (112°28′36.34″ E, 34°39′30.34″ N). In order to profile the changes in gene expression during different stages of flower development, petals from the same plant of three varieties at seven different developmental stages were sampled. These different flower developmental stages included the color−exposure stage (CE), blooming stage (BS), initial flowering stage (IF), half opening stage (HO), full blooming stage (FB), initial decay stage (ID), decay stage (DS). All samples were collected in April 2020.

The genetic transformation experiments used the seedlings of *Arabidopsis thaliana* cultivar ‘Columbia’ (Col−0) as the recipient plants, and the subcellular localization analysis used *N. benthamiana* plants. The transformed plant was cultivated in a tissue culture room (16 h:8 h, light:dark, 25 °C). The resulting transgenic plants were grown in a greenhouse (16 h:8 h, light:dark, 22 °C, 120–150 μmol·m^−2^·s^−1^ light intensity, 25–75% relative humidity).

### 4.2. Nucleic Acid Extraction and cDNA Synthesis

RNAprep Pure Polysaccharide Polyphenol plant total RNA Extraction Kit (TianGen Biotech, Beijing, China) was used to extract total RNA from the petals at different developmental stages and different tissues (including bracts, sepals, carpels, stamens, leaves, and petals) of VC, FD, and LH. The quality and concentration of nucleic acid were determined by 1% agarose gel electrophoresis and NanoDrop One (Thermo Fisher Scientific, Waltham, MA, USA). Subsequently, 100 ng of total RNA was reversely transcribed into cDNA according to the PrimeScript™ II 1st Strand cDNA Synthesis Kit Reverse Transcription Kit (TaKaRa, Osaka, Japan).

### 4.3. Cloning of PoVIN3 and Construction of Expression Vectors

Using NCBI (http://www.ncbi.nlm.nih.gov) (accessed on 25 November 2021) and the full-length transcriptome database of tree peony based on Isoform-sequencing (Iso−seq), previously constructed in our laboratory, to screen potential *PoVIN3* genes. Primer 5.0 software was used to design specific primers (Table 1), and the cDNA of the petals of FD at the full blooming stage was used as a template to amplify candidate genes. After ligation with the pMD−18T vector, the cells were transformed into *E. coli* DH5α competent cells and the positive clones were screened for sequencing verification.

Plant expression vectors pCAMBIA2300 and pCAMBIA2300−GFP were digested with restriction enzyme *Kpn* I am using correctly sequenced positive cloned plasmids as templates. The overexpression vector pCAMBIA2300-*PoVIN3* and the subcellular localization vector pCAMBIA2300−*PoVIN3*−GFP were obtained by seamless cloning technology, and the plasmid extracted from the correct bacterial solution was screened and sequenced to transform *Agrobacterium* GV3101 for subsequent tests.

### 4.4. Bioinformatics Analysis of PoVIN3

The sequencing results were analyzed by DNAMAN software. The amino acid composition of *PoVIN3*−encoded protein was analyzed by the online software Translate (http://web.expasy.org/translate/) (accessed on 10 January 2022). The physical and chemical properties, hydrophobicity, transmembrane structure, and phosphorylation sites of the protein were analyzed by ProtParam (https://web.expasy.org/protparam/) (accessed on 10 January 2022), Protscale (https://web.expasy.org/protscale/) (accessed on 10 January 2022), TMHHMM (http://www.cbs.dtu.dk/services/TMHMM-2.0/) (accessed on 10 January 2022), and NetPhos (http://www.cbs.dtu.dk/services/NetPhos/) (accessed on 10 January 2022). The conserved domains of *PoVIN3* using the NCBI Conserved Domain Database (CDD) (http://www.ncbi.nlm.nih.gov/cdd/) search tools (accessed on 10 January 2022). SOPMA (https://npsa-prabi.ibcp.fr/cgi-bin/secpred_sopma.pl) (accessed on 10 January 2022) and I−TASSER (https://zhanggroup.org//I-TASSER/) (accessed on 10 January 2022) were used to predict the secondary structure, and three-dimensional homologous modeling of the protein, respectively. Other plants *VIN3* sequences were collected from GenBank (http://www.ncbi.nlm.nih.gov/Genbank/) (accessed on 10 January 2022) and processed with default parameters using Clustal Omega (https://www.ebi.ac.uk/Tools/msa/clustalo/) (accessed on 10 January 2022) [48,49,50]. The phylogenetic tree was constructed by MEGA 7.0 software employing the Neighbor-Joining (NJ) method.

### 4.5. Analysis of Subcellular localization

The subcellular localization analysis used the 5−week−old seedlings of *N. benthamiana* plants. The constructed plasmid of pCAMBIA2300−*PoVIN3*−GFP was expanded in LB liquid medium containing 50 μg·mL^−1^ kanamycin (kan) and then transformed into the lower epidermis of *N. benthamiana* leaves by the *Agrobacterium tumefaciens*−mediated leaf disk transformation method. The infected *N. benthamiana* was incubated in the dark for 2–3 h and then incubated in the light/dark time of 16 h /8 h at 25 °C for 3 days. On the day of observation, the DAPI dye solution was injected into the back of infected *N. benthamiana* leaves, and after 3 h of culture under low light, the green fluorescent protein in leaves was observed under confocal scanning microscopy (OLYMPUS, Tokyo, Japan).

### 4.6. Arabidopsis Thaliana Transformation and Analysis of Flowering Time

The overexpression vector pCAMBIA2300−*PoVIN3* was transformed into *Arabidopsis thaliana* Col−0 via the *Agrobacterium tumefacien*-mediated method, and kan was used as a screening marker. Transgenic plants transformed with the empty vector pCAMBIA2300, and wild-type *Arabidopsis thaliana* (WT) were used as controls. The first generation (T_1_) of transgenic seedlings were screened by 50 μg·mL^−1^ kan and transplanted to the greenhouse for growth. The second generation (T_2_) of transgenic seeds was obtained by self−crossing. All transgenic lines were verified by phenotypic observation and PCR analysis.

Flowering time was analyzed by recording the number of days from the sowing of T_2_ seeds to the opening of the first flower. In addition, the data are shown as mean values ± SE (standard error). Statistical differences were analyzed by One−Way ANOVA (SPSS 19.0) at the significance level of *p* < 0.05.

### 4.7. Establishment of Transient Expression System of Tree Peony

Overnight cultures of *Agrobacterium tumefaciens* harboring pCAMBIA2300 empty vector and pCAMBIA2300−*PoVIN3* were grown at 28 °C in liquid LB medium supplemented with 50 μg·mL^−1^ kan and 20 μg·mL^−1^ Rifampin. *Agrobacterium tumefaciens* cells were centrifuged at 6000 rpm for 10 min at 4 °C and suspended in the infiltration buffer composed of 10 mM MgCl_2_, 200 mM acetosyringone, and 10 mM MES/KOH pH 5.6 to a final optical density reached 1.8–2.0 at 600 nm.

Tree peony petals were collected from *Paeonia suffruticosa* ‘Luoyanghong’ (Petals purplish red, base with inky purple spots) at HO. the outermost whorl of the flowers was discarded, and the petals were divided into two forms: whole petals and disc for subsequent processing. Using a hole punch to remove a point five−centimeter−diameter disc from the center of the petals. The fresh tree peony petals or discs were randomly divided into several equal parts, wrapped with gauze, and immersed in different bacterial suspension solutions. For vacuum infiltration, tree peony petals or discs were infiltrated under vacuum at 0.07 MPa for 10 min. After releasing the vacuum, the petals and discs were washed four times in deionized water and placed in sterile petri dishes (9 cm in diameter) with 5–10 mL of deionized water, respectively. Subsequently, they were held at 8 °C for 2–3 days, then at 23 °C for 1 day. The tree peony petals and discs were dehydrated for 12 h and then placed in deionized water for rehydration for 12 h. The relative length, relative width, and relative fresh weight of all petals and discs were determined at 3 h intervals. qRT−PCR was used to measure the relative expression levels of the target gene.

Similarly, the flowers of *Paeonia suffruticosa* ‘Luoyanghong’ at BS with 5 cm pedicels were cut off and placed in 40 mL of bacterial suspension solution, and the buds were wrapped with gauze and soaked in 10 mL of bacterial suspension solution. The buds were vacuumized to 0.07 MPa for 30 min and then slowly exhaled. There were 5 flowers per treatment, single flower repeat. The aspirated petals were rinsed with deionized water to remove excess bacterial suspension solution. The pedicels were still immersed in the corresponding bacterial suspension solution and cultured in the dark at 8 °C for 2–3 days, then transferred to 23 °C for 1 day. Then, the fresh-cut tree peony flowers were cultured under normal light and sampled every 7 h. The relative expression levels of target genes and the flowering time of fresh−cut flowers of tree peonies were detected compared with the control.

### 4.8. Quantitative Real−Time PCR Analysis of PoVIN3

Total RNA samples collected from different tissues and petals of developmental stages were used to analyze the spatial and temporal expression patterns of *PoVIN3*, and the expression levels were investigated by quantitative real−time PCR (qRT−PCR). The tbulin-α gene of tree peony was used as the reference gene. Specific primers for quantitative real−time PCR (qRT−PCR) were designed according to the CDS sequence of *PoVIN3* (Table 1). The qRT-PCR was performed on the Light Cycler 96 System (Roche, Germany) using TB Green Premix Ex Taq II (Takara, Japan). Each sample was assayed in four biological replicates, and relative expression levels were analyzed using the 2^−ΔΔCT^ method [51]. SPSS 19.0 and Origin 8.5 software was used for statistical analysis. Duncan’s multiple comparison tests were used to determine significant differences between samples. Error bars in all figures represent the standard error from the mean.

## 5. Conclusions

In conclusion, *PoVIN3*, a key gene of the vernalization pathway, was first identified and described in *Paeonia ostii* ‘Fengdan’. The current findings have revealed details about the potential features of *PoVIN3*. *PoVIN3* was the highest at the initial flowering stage of the variant cultivar of *Paeonia ostii* ‘Fengdan,’ the initial decay stage of the normal flowering *Paeonia ostii* ‘Fengdan,’ and the half opening stage of the late flowering *Paeonia suffruticosa* ‘Lianhe.’ Heterologous overexpression of *PoVIN3* in *Arabidopsis Thaliana* further clarified its functions. The flowering time was advanced in transgenic *Arabidopsis thaliana* plants hosting *PoVIN3*. *PoVIN3* may act as positive regulatory factor affecting flowering time, thus promoting plant flowering.

## Figures and Tables

**Table 1:** Primer sequence in this study.

Primer Name	Sequence (5′-3′)	Use
*PoVIN3*	F: ATGACGGATAATCCAAAGAC	Cloning the full−length of ORF
	R: TTAATGCCATAGCTTACTGC	
*PoVIN3*−*qRT*	F: CAGGGATACTCGGGGTTT	Quantitative Real−time PCR
	R: TCTGCTGACAATGCCACG	
*Tbulin-α*	F: CCGTCAACTTTTCCACCCTG	
	R: CCTCACTCGGTCAAGGCAGA	
pCAMBIA2300−*PoVIN3*−GFP	F: GGAGAGGACAGGGTACCATGACGGATAATCCAAAGAC	Subcellular localization
	R: GATCCCCGGGTACCATGCCATAGCTTACTGCAGAAC	
pCAMBIA2300−*PoVIN3*	F: GGAGAGGACAGGGTACCATGACGGATAATCCAAAGAC	
	R: GGATCCCCGGGTACCTTAATGCCATAGCTTACTGC	
*AtVIN3*	F: CCGTAAAGACTGGCGAACAG	Functional verification
	R: CCGCACGAGTAACCCTGAT	

## Data Availability

The data supporting the results are already mentioned in the main text.
